# Cultured Cells Isolated from CNS Indolent B-Cell Lymphoma Have Characteristics of Mesenchymal Stem Cells: A Clinical Case and Scientific Research

**Published:** 2018-07-01

**Authors:** Kalina Tumagelova-Yuzeir, Ekaterina Ivanova-Todorova, Emanuil Naydenov, Tsvetelina Velikova, Ekaterina Krasimirova, Kalina Belemezova, Tihomir Dikov, Marin Penkov, Sevdalin Nachev, Margarita Genova, Dobroslav Kyurkchiev

**Affiliations:** 1 Laboratory of Clinical Immunology, University Hospital “St. Ivan Rilski”, Department of Clinical Laboratory and Clinical Immunology, Medical University of Sofia, Sofia, Bulgaria; 2 Clinic of Neurosurgery, University Hospital “St. Ivan Rilski”, Sofia, Bulgaria; 3 Laboratory of Neuropathology, University Hospital “St. Ivan Rilski”, Sofia, Bulgaria; 4 Laboratory of Hematopathology and Immunology, National Specialized Hospital for Active Treatment of Hematological Diseases, Sofia, Bulgaria; 5 Department of CT and MRI, University Hospital “St. Ivan Rilski”, Sofia, Bulgaria; 6 Tissue Bank “BulGen”, Sofia, Bulgaria

**Keywords:** CNS lymphoma, Indolent B-cell lymphoma, Mesenchymal stem cells, Immunomodulation

## Abstract

The case report presented here describes the culturing and characterization of mesenchymal stem cells (MSCs) isolated from a primary indolent B-cell lymphoma, located in the CNS of an immunocompetent patient. The presence of such cells in the tumor mass can further elucidate the pathogenesis of the disease and reveal possible future approaches for its treatment. We present a case report of a 61-year-old immunocompetent woman who had an episode of confusion with numbness in the right leg and the right arm, slurred and dysarthric speech and urine incontinence. The peripheral blood tests were normal. The neurological examination demonstrated a latent hemi-paresis of the right side, aphasia, discrete hypertension and bradypsychia. The ophthalmologic examination revealed left quadranopsia. Computed tomography and magnetic resonance imaging of the brain showed a 3.5 × 2.9 cm infiltrative neoplastic lesion involving the left temporal parenchyma. The morphological features and the immunophenotyping of the lymphoid cell composition were consistent with low-grade (indolent) B-lymphocyte non-Hodgkin’s lymphoma of CNS. Cells, isolated from the resected tumor mass, were cultured *in vitro* in medium containing 10% fetal bovine serum (FBS) and characterized by their morphology, growth, phenotype, clonogenicity and osteogenic differentiation. It was apparent that the cultured cells isolated from the indolent B- cell lymphoma located in the CNS have the basic characteristics of mesenchymal stem cells. The presence of MSCs is described for the very first time in such type of tumor. The well-known immunosuppressive properties of the MSCs may represent another mechanism favouring the tumor growth.

## Introduction

 A rare detection of non-Hodgkin’s lymphoma, presented as an intracranial mass lesion confined to the central nervous system (CNS) at the time of diagnosis, is defined as a primary CNS lymphoma^1^. The vast majority of primary CNS lymphomas in immunocompetent patients are diffuse and large B-cell lymphomas, and very rarely they are T-cell lymphomas or low grade (indolent) B-cell lymphomas^2^. The incidence of B-cell lymphomas is reported to be up to 3-4% of all primary brain tumors and approximately accounts for 3–20% of all primary CNS lymphomas. Some studies suggest progressively increasing occurrence of B-cell lymphomas in immunocompetent patients^1^. Generally, the indolent B-cell lymphomas involve the dura and spare the cerebral tissue. Unlike many primary brain tumors, low-grade primary CNS lymphomas may exhibit a more favourable prognosis than high-grade ones. The indolent B lymphomas are typically responsive to treatment, which may lead to remission or complete cure of the patient^2^. Furthermore, low-grade primary CNS lymphomas may be treated less aggressively, with treatment limited to the tumor region (i.e., local radiation therapy or surgery) and with a lower risk of late neurotoxicity^2^. 

## Case presentation

**Figure 1:**
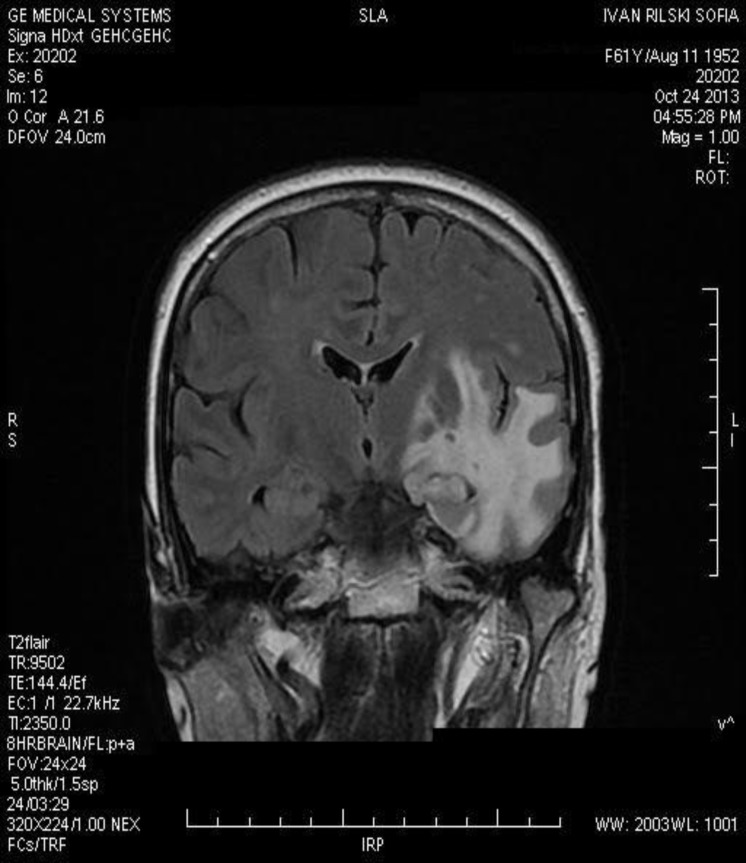
MRI image of the brain. It shows an infiltrative neoplastic lesion involving left temporal parenchyma

We present a 61-year-old immunocompetent woman. A month prior to her hospital admission, she had an episode of confusion with numbness in the right leg and arm, slurred and dysarthric speech. She reported urine incontinence during the night that had occurred once during the past month. There was no data for lymphoma in the family. Peripheral blood tests were normal. The neurological examination demonstrated a latent hemi-paresis of the right side, aphasia, discrete hypertension and bradypsychia. The ophthalmologic examination revealed left quadranopsia. Computed tomography and magnetic resonance imaging of the brain showed a 3.5 × 2.9 cm infiltrative neoplastic lesion involving the left temporal parenchyma (Figure 1). 

Based on clinical and MRI data, the patient underwent left frontо-temporal craniotomy with partial excision and verification of the tumor. The lesion was surgically excised under sonographic control in the conditions of intra-operative contrast enhancement with Fluorescein Na. After the operation, the patient demonstrated satisfactory state with persistent bradypsychia and discrete aphasia. The patient was administered on Depaquin 0.5 mg daily and recovered well after the surgical intervention. Histological examination of the resected tumor described lymphoid tumor cells with small oval hyperchromic nucleus, condensed chromatin and scarce cytoplasm. The tumor cells were located in the peri-vascular spaces, arranged in concentric pattern among layers of reticulin-positive (Gomory +) material around blood vessels. There were significant reactive changes in the adjacent brain tissue. The neoplastic lymphoid cells were positive for CD45, CD20 and Bcl-2, whereas they were negative for CD5, CD10, CD23, Bcl-6, and Cyclin D1 as determined by immunohistochemistry, with a very low proliferation rate of 1% revealed by Ki-67, admixed with numerous reactive CD3+CD5+ T cells. The morphological features and immunophenotyping of lymphoid composition were consistent with low-grade (indolent) B-lymphocyte non-Hodgkin’s lymphoma of CNS. After determination of the stage of the disease, chemotherapy was administered, but the patient acquired pneumonia and died after ten months.

## Discussion

 We present a very rare case of indolent B-cell lymphoma, arising exclusively intraparenchymal primary in CNS. Malignant lymphocytes of CNS lymphomas usually freely invade normal surrounding brain and infiltrate dural membrane. Our case is a unique example of indolent B-cell lymphoma in which there was no dural involvement, and the lesion was exclusively intraparenchymal. Moreover, this low grade B-cell primary CNS lymphoma was presented in an immunocompetent patient. Here, we report for the first time the characterization of cultured mesenchymal stem cells, isolated from indolent B-cell lymphoma arising exclusively intra-parenchymal, primary in CNS of an immunocompetent patient. The patient signed informed consent documents according to the requirements of the Ethics Committee of University Hospital “St. Ivan Rilski”, Sofia, Bulgaria.

**Morphology and phenotype**

**Figure 2:**
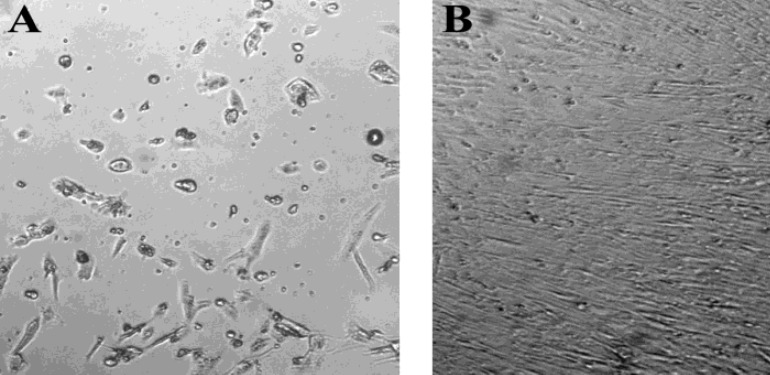
Morphology of cultured cells isolated from indolent CNS lymphoma.The cells cultured in medium containing FBS adhere to the plastic and initially they are rather heterogeneous population (A) but after 15-18 days the cells form a monolayer consisting of elongated fibroblast-like cells (B).

The lymphoma isolated cells, cultured in medium containing 10% FBS, showed a growth pattern very similar to that of MSCs characterized by initial heterogeneity of the morphology (Figure 2A), and at later stage (15- 18 days in culture) the cells had the typical fibroblast-like morphology. The cells adhered to the solid phase and formed a confluent monolayer (Figure 2B).

**Figure 3:**
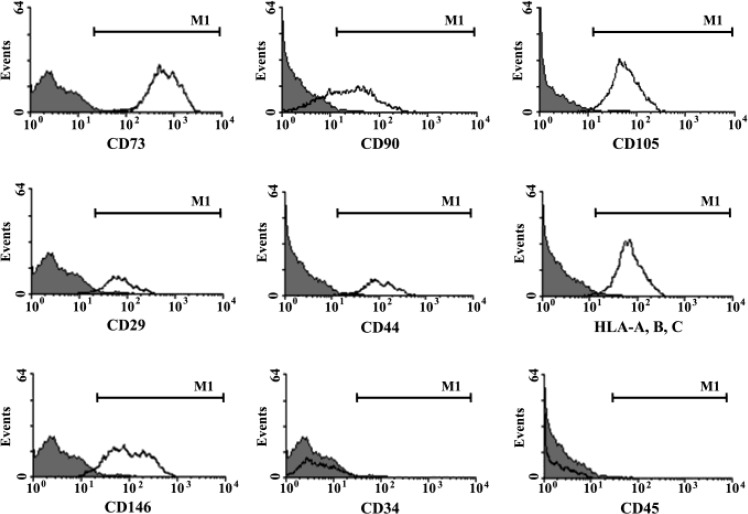
Phenotype characteristics of cultured cells isolated from indolent CNS lymphoma.More than 90% of the cultured cells express CD73, CD90, CD105, CD29, CD44, HLA-I (A, B, C), CD146 but no expression of CD34 and CD45 was recorded.

When the culture was analysed by flowcytometry, it was established that the cells expressed the same markers that had all characteristics of the MSCs, including CD90, CD73, CD105, CD29, CD44, HLA-I (A,B,C) and CD146, but were negative for CD45 and CD34 again just like the MSCs (Figure 3). 

**Clonogenicity**

**Figure 4:**
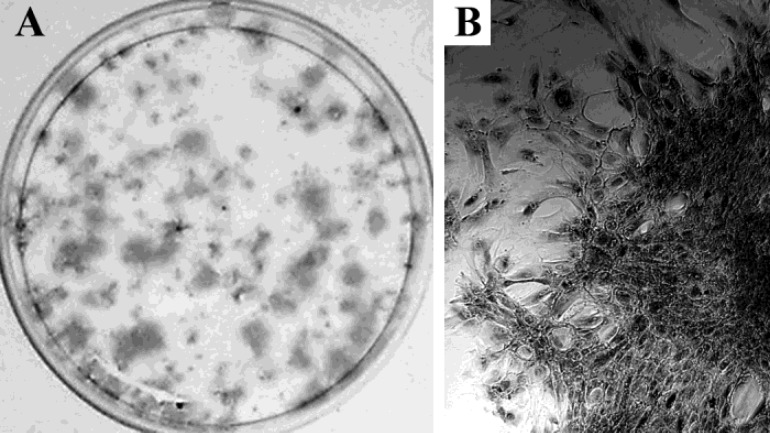
Clonogenic capacity of cultured cells isolated from indolent CNS lymphoma. The isolated cells demonstrate a colony formation capacity. A - Plate with Crystal Violet-stained colonies observed after 14 days of cultivation of the cells. B - Representative colony (magnification x 100).

The ability of stem cells to form clones is an expression of their basic property – self-renewal. Any cell that lacks such capacity cannot be defined as a “stem cell” and this requirement is present in any definition of stem cells. The results from our experiments in testing the clonogenicity of cultured cells showed that these cells formed well-defined colonies when seeded at density of 300 cells/cm^2 ^and cultured for 14 days. The dimensions of the colonies were in the ranges of 0.5 – 2.5 mm in diameter, as the smallest ones were composed of 20-25 cells (Figure 4). 

**Osteogenic differentiation**

**Figure 5:**
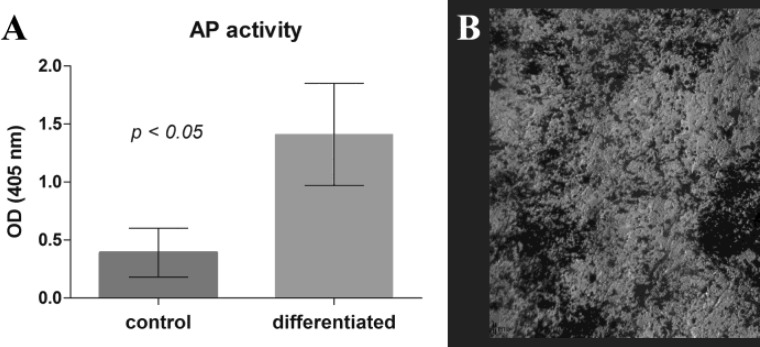
Osteogenic differentiation potential of cultured cells isolated from indolent CNS lymphoma. Osteogenic differentiation was quantitatively measured by alkaline phosphatase assay (A) and demonstrated by calcium deposition visualized by von Kossa staining (B).

The osteogenic differentiation is a typical feature of MSCs and was shown by the capacity of the cells to express intracellularly alkaline phosphatise. When cultured in medium containing osteogenic inductive factors, lymphoma isolated cells had significantly higher alkaline phosphatase activity as compared to the undifferentiated control cultures (Figure 5A). The osteogenic differentiation of the cells was further confirmed by von Kossa staining (Figure 5B).

**Cytokine secretion**

The conditioned medium of cultured cells was tested for the secretion of 11 basic immunoregulatory factors (IL-6, IL-10, IL-12, TNFα, IFNγ, IL-8, IL-18, IL-17A, IL-23, sPECAM, sICAM) and a highly increased concentration of IL-6 (111.05 pg/ml) and IL-8 (848.1 pg/ml) were detected in comparison with the levels in the control medium. No secretion was detected for the rest of the cytokines.

Having in mind the classical definition of MSCs as cells adherent to plastic, with fibroblast-like morphology and potential for clonogenicity and differentiation into mesenchymal lineages^3^, it can be assumed that the cultured cells, isolated from indolent CNS lymphoma, in fact represent MSCs. Moreover, they express phenotypic markers typical for MSCs^4^, and secrete cytokines such as IL-6 and IL-8 which are engaged in immunoregulatory activities expressed by the MSCs ^5^.

The presence of MSCs in the CNS has been recently described. In 2012, the group of Paul reported the presence of multipotent progenitor cells in the peri-vascular spaces in CNS of adult people ^6^. МSCs can be found not only in normal brain tissue but also in tumors of CNS as well. For instance, the cells isolated from Glioblastoma multiforme (GBM), the most common brain malignancy, and cultured in medium with FBS demonstrate typical characteristics of MSCs. The authors claim that the so called “adherent cells” (GBM-ACs), which are a product of culturing of GBM isolated cells, are in fact MSCs. In support of their assertion, the authors have found in GBMs *in vivo* presence of cells located in the peri-vascular sites, which have phenotype of MSCs proved by expression of CD105, ADAM12, PDGFRβ, CD73, CD90 and negative expression for CD31 and NG2^7^. Hossein et al. express their opinion that MSCs are an important part of the stroma of GBM and the *in vitro* results from experiments with GBM-ACs (equivalent to the MSCs according to the authors) reveal that these cells stimulate the proliferation, tumorigenicity and the self-renewal potential of the tumor cells through the IL-6 / STAT3 system. On the other hand, there is data claiming that by using CCL-2, МSCs are able to impair the invasion and growth of GBM cells^8^. 

There are few and contradictory results in the literature about the interaction of lymphoma cells with MSCs. Thus, aiming to establish the effect of the MSCs on the survival of lymphoma xenografts, the group of Secchiro injected i.p. SCID mice with lymphoma cell line (BJAB/SKW6.4), and 4 days later the MSCs were injected in the lymphoma itself. The mice treated with BJAB/SKW6.4 and MSCs demonstrated significant increase of the survival rate in comparison to mice injected with lymphoma xenograft only. The same study clearly demonstrated that *in vitro *co-culturing with MSCs influences the survival/proliferation of the lymphoma cell lines ^9^. 

However, there are data on the effects of MSCs on malignant cells, which is contrary to those cited above. Thus, experiments with follicular lymphomas revealed that under the effects of MSCs the survival of the malignant B cells was stimulated ^10^, and MSCs via the secretion of CCL-2 recruit monocytes to the tumor and later transform them in population, similar to the tumor-associated macrophages^11^. Data from other studies show that the interactions of myeloma cells with MSCs isolated from the tumor enhance the proliferation of the myeloma cells in comparison to the effects of MSCs, isolated from healthy humans ^12^.

One of the functions of the MSCs present in the indolent CNS lymphoma could be associated with the suppressive effect of these cells on the immune system. The most important immunosuppressive effect of the MSCs seems to be associated with their effect on the dendritic cells (DCs). DCs, which are under the effect of the MSCs, are characterized by immature tolerogenic phenotype manifested by a lower expression of MHC class II antigens and co-stimulatory molecules, and an increased secretion of IL-10. The antigen presentation performed by these modulated DCs causes the induction of energy in the T lymphocytes ^13^. IL-6 secreted by the MSCs are of particular importance for the formation of tolerogenic DCs, and it has been shown that IL-6 modulates the STAT-3 pathway which results in lower expression of MHC class II products and the B7 co-stimulatory complex ^14^. As mentioned above, the MSCs isolated from the indolent CNS lymphoma were shown to secrete high amount of IL-6. 

Concerning the secretion of IL-8 by the cells cultured in our experiment, it can be speculated that its role is related to attraction of immunosuppressive cells, having in mind the suppressive function of the MSCs in immunoregulation.

## CONCLUSION

 In conclusion, our results for the first time report the presence of MSCs in indolent lymphoma with CNS localization, and this puts the grounds to discuss some new aspects of the disease and possible new therapeutic approaches. 
